# Structural analysis of women’s social representations of urinary incontinence

**DOI:** 10.1590/1980-220X-REEUSP-2024-0433en

**Published:** 2025-07-07

**Authors:** Manuela de Mendonça Figueirêdo Coelho, Amanda Holanda de Mesquita, Beatriz Alves de Oliveira, Camila Barroso Martins, Riksberg Leite Cabral, Mônica Oliveira Batista Oriá, Mariana Cavalcante Martins, Janaína Fonseca Victor Coutinho, Fabiane do Amaral Gubert

**Affiliations:** 1 Universidade Federal do Ceará, Fortaleza, CE, Brazil.; 2 Prefeitura Municipal de Maracanaú, Maracanaú, CE, Brazil.

**Keywords:** Urinary Incontinence, Enterostomal Therapy, Social Representation, Women’s Health

## Abstract

**Objective:**

To assess the structure of social representations of urinary incontinence among Brazilian women.

**Method:**

A cross-sectional study, which used semi-structured questionnaires applied online between September 2021 and June 2022. A total of 796 Brazilian women participated. Lexical and similarity analyses were performed using the EVOC 2000^®^ and IRAMUTEQ^®^ software. Evoked words were organized based on frequency and evocation hierarchy.

**Results:**

The analyses indicated that the words “urine”, “embarrassment”, “pee”, “discomfort”, “pain”, “discomfort”, “urine leakage”, “infection”, “bladder”, “burning”, “leakage”, “fear”, “wet”, “problem” and “disease” were at the core of social representations about urinary incontinence. These terms were frequently mentioned and appeared primarily in the responses. In addition to the physical impact, urinary incontinence was associated with social isolation, anxiety and negative effects on sexual health.

**Conclusion:**

Urinary incontinence affects several aspects of women’s lives, imposing negative representations in both physical and emotional dimensions. This study highlighted the urgency of destigmatizing the condition and promoting more comprehensive care approaches that consider the complexities of these social representations.

## INTRODUCTION

Urinary incontinence (UI) is a global public health condition that affects between 20% and 45% of women, depending on the population group and the diagnostic criteria used^(1)^. Characterized by involuntary loss of urine, it impacts multiple dimensions of women’s lives, encompassing physical, emotional and social aspects.

It can be classified into three main types: stress UI, associated with activities that increase intra-abdominal pressure; urge UI, linked to detrusor muscle overactivity; and mixed UI, which combines features of stress UI and urge UI, resulting in greater severity of symptoms. The condition is often neglected and stigmatized, which exacerbates the challenges for early diagnosis and appropriate treatment^(2)^.

The impacts of UI go far beyond physical limitations, such as urinary tract infections, reduced mobility and discomfort. Studies show that the condition is associated with anxiety, depression and social isolation, which profoundly affect women’s quality of life and mental health^(3,4,5)^. Additionally, many women avoid discussions about UI, do not seek treatment, or minimize their condition due to stigma and cultural barriers, exacerbating feelings of shame and embarrassment^(6)^. In the sexual sphere, UI can compromise physical intimacy, resulting in sexual dissatisfaction and deterioration of marital relationships^(7,8)^.

Nursing practice is essential in UI prevention and management in women, contributing to the early identification of symptoms and the implementation of effective interventions. Care includes education on pelvic floor exercises, guidance on voiding habits, and emotional support to mitigate the psychosocial impacts of UI, promoting patients’ quality of life^(9)^.

The specialty of enterostomal therapy, recognized as an advanced area of nursing practice, is broadly trained in the care of conditions such as UI. Enterostomal nurses have specialized training to address the complex demands associated with UI, including clinical management, prevention of complications, and emotional support for patients.

Although the medical and epidemiological literature addresses UI, the way women attribute meanings to UI and interpret its consequences is still incipient, although it is fundamental to understanding behavior in the face of the disease and adherence to treatments. The Social Representation Theory (SRT), proposed by Moscovici^(10)^ and expanded by Abric^(11)^, provides a robust analytical framework to explore the meanings attributed to health conditions, such as UI, and their implications for social practices.

SRT postulates that social representations are forms of knowledge that are collectively constructed and shared, resulting from daily experiences and social interactions. These representations fulfill two primary functions: making the unfamiliar familiar and guiding behaviors. The theory’s structural approach^(11)^ suggests that social representations have a central core, composed of stable and widely shared elements, and a peripheral system, which is more flexible and adaptable to the context. In the case of UI, the central core of representations may be associated with feelings of shame and discomfort, whereas the peripheral system may reflect more subjective experiences, such as coping strategies and individual perceptions.

The relevance of exploring social representations of UI lies in understanding how women perceive and experience this condition. Representations reflect subjective experience of UI and influence social practices, from seeking clinical care to discussing the condition in private and public contexts. However, there is a gap in current literature, with few studies focusing on UI from this perspective, especially in Brazil, where cultural and social factors play a relevant role in the management of this condition^(12,13)^.

This study seeks to fill this gap by providing a detailed analysis of Social representations of UI among Brazilian women. The use of SRT allows us to identify the central and peripheral elements of these representations, providing support for the formulation of public health strategies that address the emotional, social, and physical needs of women affected by UI. Moreover, the results of this study can directly contribute to nursing practice by guiding nurses in the development of interventions that promote health education, prevention of complications, and emotional support for women. This perspective strengthens the role of nurses as agents of transformation in comprehensive women’s healthcare. Thus, this study aimed to assess the structure of Social representations of UI among Brazilian women.

## METHOD

### Study Design

This research adopts a qualitative approach with a theoretical- methodological framework guided by SRT, with emphasis on its structural aspect proposed by Moscovici^(10)^ and Abric^(11)^. Within the scope of SRT, it is postulated that collective expression is structured around a central core, which encapsulates social memory, and peripheral elements, which reflect the immediate and adaptable context. This configuration allows for an in-depth understanding of the object of study and the relationships that individuals establish with perceived reality as well as their integration into the cognitive universe. Thus, the theory seeks to elucidate the connection between subject and object, outlining social representations rooted in common sense. The organization of information in this manuscript was guided by COnsolidated criteria for REporting Qualitative research recommendations.

SRT’s structural approach is characterized by presenting social representations as a dual system. The central core is composed of a restricted set of stable ideas that are widely shared by group members, demonstrating resistance to change. The peripheral system, which is more flexible and sensitive to the immediate context, derives its meaning and function from its relationship with the central core^(11,14)^. The peripheral system performs multiple functions, including central core concretization through positioning or conduct, the prescription of behaviors and personalized modulation. The latter allows a representation to manifest itself in apparently distinct ways, reflecting individual appropriations or specific contexts, always in line with the central core^(10,11)^.

### Place, Population, Selection Criteria

Data were collected between September 2021 and June 2022, covering a universe of Brazilian women, who represent more than half of the national population. Collection was carried out for convenience, resulting in the participation of 796 women, who answered a semi-structured questionnaire made available online. The invitation to participate in the survey was disseminated through several social media platforms, including groups aimed at women from different regions of Brazil. In addition, participants were asked to share the survey link with other women on their contact lists, expanding the reach of collection.

Potential participants, defined as Brazilian women over 18 years of age, received detailed information about the study and expressed their interest in participating in the research by responding to the call for proposals posted on social media or by sending a link to other participants. Furthermore, they received explanations about the purpose of the research and how to participate. After accepting, all participants signed the Informed Consent Form and received a link to access the questionnaire. The questionnaire included sociodemographic, clinical and obstetric data, in addition to the central question “Tell me what comes to mind immediately when you think of UI”, with spaces for writing up to three words or expressions in the order in which they appeared. Women who did not present the three requested evocations about UI were excluded.

### Data Analysis

Sociodemographic, clinical and obstetric data were consolidated in Excel^®^ and exported for analysis in the Statistical Package for the Social Sciences version 23.0 and presented using descriptive statistics, with absolute and relative frequencies and standard deviations.

To triangulate data from the text *corpus*, the qualitative data from evocations about what women thought about UI were subjected to lexical and content analyses. The recorded words were exported to Word^®^ format files, coded and standardized in terms of gender and number inflections, and later saved in .txt format, compatible with the *Ensemble de Programmes Permettant l’Analyse des Évocations* (EVOC 2000^®^) software. This software is specialized in constructing the Four-Quadrant Chart, which represents the representation’s central and intermediate cores.

The frequency of words and the Average Order of Evocations (AOE) were considered using the following parameters: minimum frequency of 05; intermediate of 10; and Rang of 2.5^(15)^. EVOC 2000^®^ has generated the Four-Quadrant Chart, which organizes evocations into four zones based on frequency and AOE:

- Central core (upper left quadrant): includes high frequency and low AOE terms, representing the most stable and important elements for the group;
- First periphery (upper right quadrant): comprises high frequency elements and greater AOE, indicating lesser relevance in the immediate imagination;
- Contrast zone (lower left quadrant): contains low-frequency, rapidly recalled words, reflecting ideas relevant to specific subgroups;
- Second periphery (lower right quadrant): represents low frequency and high AOE elements, considered less important or marginal in social representation.

Subsequently, the Descending Hierarchical Classification was performed in the *Interface de R pour les Analyses Multi­dimensionnelles de Textes et de Questionnaires* (IRAMUTEQ^®^), segmenting the text *corpus* into hierarchically organized classes, highlighted in the dendrogram. Each class is composed of words with a high c^2^ value, indicating a strong association with the category. The hierarchical structure allows the identification of thematic relationships among elements, revealing emerging patterns in the text *corpus*.

Similarity analysis, also performed in IRAMUTEQ^®^, identified the co-occurrence relationships among evoked terms. The results were represented graphically by a similarity tree^(16)^, in which:

- Main nodes: represent the most frequently connected terms, such as “shame” or “pain”;
- Connections: the thickness of edges indicates the intensity of the association among terms;
- Secondary branches: identify subgroups of connected words, helping to interpret nuances of social representations^(16)^.

### Ethical Aspects

The study was approved by the Research Ethics Committee under Opinion 4,270,415. All participants signed the Informed Consent Form, guaranteeing confidentiality and voluntariness. The data from this research are available on the OSF Home through the link https://osf.io/79c2u/.

## RESULTS

The women who participated in the study had an average age of 34.6 years (SD = 12.4), with 333(43.3%) white, with a partner (n = 482, 60.8%) and an average weight of 69.8 kg (SD = 23). Among the participants, 531(66.7%) worked; 473(59.4%) studied; 202(25.4%) had incomplete higher education; 188(23.6%) higher education and specialization; and 174(21.9%) had only higher education.

Concerning clinical and obstetric characteristics, 371(46.6%) were pregnant, with an average of 0.96 (SD = 1.2) pregnancies and an average of 0.83 (SD = 1.1) normal deliveries. At the time of the survey, 82.8% (n = 659) reported being in their reproductive period. Regarding knowledge about the existence of exercises to strengthen the pelvic floor, 673(85.5%) stated that they knew about the existence of pelvic floor strengthening practices, but 604(75.9%) did not practice the exercises.

Using the 3,533 evocations, the Four-Quadrant Chart (Chart 1) was constructed.

**Chart 1:** Structure of women’s social representation of urinary incontinence – Fortaleza, Ceará, Brazil, 2022.

Central elements			Intermediate elements		
Frequency ≥ 10		Rang < 2.5	Frequency ≥ 10		Rang > 2.5
Evocation	Freq*	Rang	Evocation	Freq*	Rang
Urine	148	1.574	Treatment	19	2.737
Shame	128	2.148			
Pee	128	1.414			
Nuisance	113	1.593			
Pain	103	1.650			
Discomfort	83	1.892			
Urine loss	69	1.304			
Infection	63	1.905			
Bladder	37	1.946			
Burning	32	1.969			
Leakage	29	1.483			
Fear	29	2.345			
Wet	27	2.444			
Problem	25	2.240			
Disease	23	2.348			
**Intermediate elements**			**Peripheral elements**		
**Frequency < 09 ≥ 05**		**Rang < 2.5**	**Frequency < 09 ≥ 05**		**Rang < 2.5**
**Evocation**	Freq*	**Rang**	**Evocation**	Freq*	**Rang**
Cough	09	2.444	Smell	06	2.500
Anxiety	08	2.375	Healing	06	2.667
Spontaneous	06	2.333	Stress	06	3.000
Loose	08	2.125	Musculature	06	2.833
Disability	08	2.125	Absorbent	05	2.800
Urgency	08	2.000	Surgery	05	2.600
Dysfunction	07	2.000	Hygiene	05	2.600
Hurry	05	2.000	Medical	05	2.600
Bad	05	2.000	Medicine	05	2.800
Low-bladder	09	1.889			
Muscles	07	1.857			
Suffering	05	1.8			
Worry	07	1.714			
Pregnancy	06	1.333			

Note: *Frequency.

The results of the Four-Quadrant Chart highlighted that the central nucleus of Social representations of UI is made up of 15 high-frequency words and low AOE. Among them, words such as “urine” (148 times; AOE 1.574), “shame” (128; AOE 2.148), “pee” (128; AOE 1.414), “discomfort” (113; AOE 1.593) and “pain” (103; AOE 1.650) reflect both the physical symptoms and the negative emotions associated with the condition. These terms reinforce the perception of UI as an uncomfortable and emotionally draining physical experience. The word “fear” (29; AOE 2.345) emerges as a relevant element connected to the fear of embarrassing situations. Words associated with the physical dimension, such as “infection” (63; AOE 1,905) and “leakage” (29; AOE 1,483), also appear prominently, reflecting concerns about the clinical consequences of UI.

In the first periphery, terms related to potential solutions were identified, such as “treatment” (19; AOE 2.737). In the second periphery and in the contrast zone, words such as “anxiety” (8; AOE 2.375), “hurry” (5; AOE 2.000) and “suffering” (5; AOE 1.800) illustrate more subjective and subgroup- specific perceptions.

**Figure 1:**
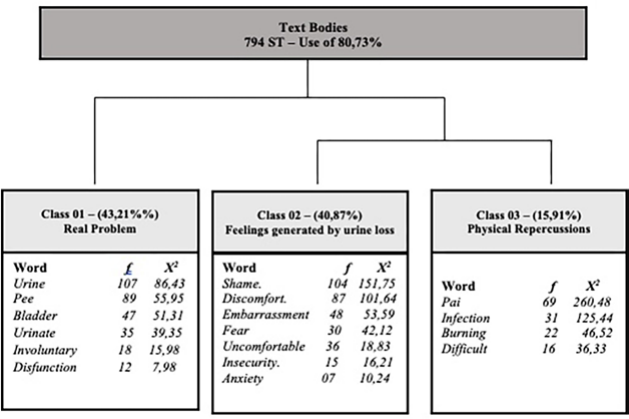
Descending hierarchical classification of women’s evocations of urinary incontinence – Fortaleza, Ceará, Brazil, 2022.

In the Descending Hierarchical Classification analysis, the *corpus* consisted of 796 texts, separated into 794 text segments (TSs), with 691 TSs (87.03%) being used. There were 3,533 occurrences (words, forms or words). The content analyzed was categorized into three classes (Figure 1):

- Class 1 – Real problem (40.96%): includes words such as “urine” (f = 107; c^2^ = 86.43), “pee” (f = 89; c^2^ = 55.95) and “bladder” (f = 47; c^2^ = 51.31), confirming the relevance of the central symptoms in the participants’ experience.
- Class 2 – Feelings generated by urine loss (14.76%): includes terms such as “shame” (f = 104; c^2^ = 151.75), “discomfort” (f = 87; c^2^ = 101.64) and “fear” (f = 30; c^2^ = 42.12), highlighting the emotional and social impact of UI.
- Class 3 – Physical repercussions (44.28%): focuses on clinical consequences and symptoms, such as “pain” (f = 69; c^2^ = 260.48), “infection” (f = 31; c^2^ = 125.44) and “burning” (f = 22; c^2^ = 46.52), which impact women’s quality of life.

The interlocution of classes converges when class 1 discusses incontinence as a real problem, generating feelings and physical repercussions that, according to the person assessed, have a negative connotation for the woman’s life. There was a confluence of words presented in the three classes and in the possible central core of social representations.

**Figure 2:**
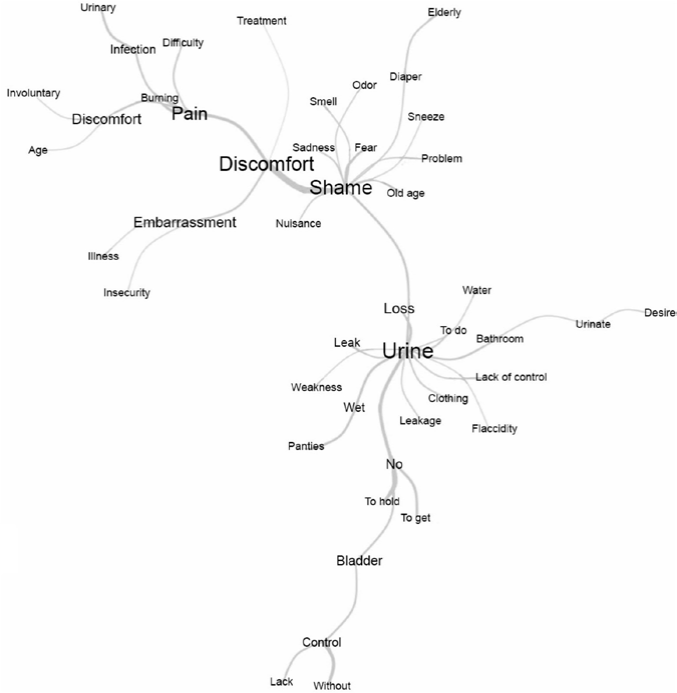
Similarity analysis of women’s evocations about urinary incontinence – Fortaleza, Ceará, Brazil, 2022.

To better explore the collected materials, a similarity analysis was performed. Through analysis based on graph theory, it was possible to identify the textual occurrences between words and indications of connectivity between them, helping to identify the content structure of a text *corpus*. Similarity analysis of evocations produced the maximum tree (Figure 2).

The analysis confirms the representation cores indicated in Chart 1 and the interconnection between the representative characteristics of UI, such as “pee”, as well as the feelings indicated by women, such as “shame” and “discomfort”, in addition to the lexicon “pain”, such as a physical repercussion.

The term “urine” is connected to “leak”, “loss” and “leakage”, reinforcing the association with the loss of urinary control and represents an immediate and concrete perception of UI, being widely connected to words that describe the physical symptoms and challenges associated with urinary control. This node reflects the central manifestation of the condition, standing out for its simplicity and universality in women’s vocabulary.

The “discomfort” node is connected to the words “wet”, “insecurity”, “burning”, “embarrassment”, “leakage”, anxiety”, among others. It occupies a central place in the similarity tree, representing both the physical and emotional dimensions of UI experience. It connects to other terms that reflect the direct impacts of the condition on women’s daily lives and quality of life.

“Shame” is related to feelings such as “fear”, “sadness” and “embarrassment”, highlighting the emotional impact of the condition. The “shame” node stands out as one of the central emotional pillars in similarity analysis, reflecting the symbolic and stigmatizing burden associated with UI. This node presents significant connections with terms such as “discomfort”, “fear”, “sadness”, “embarrassment” and “insecurity”, which together draw a dense and multifaceted emotional picture.

The “pain” node reflects the physical impact of UI and is linked to “discomfort”, “burning”, “infection”, “difficulty” and “suffering”. These terms highlight the physical repercussions that aggravate daily limitations and affect women’s quality of life (Figure 2).

## DISCUSSION

The results of this study support the complexity of UI as a condition that profoundly impacts the physical, emotional and social dimensions of women’s lives. The structural analysis of social representations revealed central, intermediate and peripheral elements that support the shared experiences of UI. Words such as “urine”, “shame”, “pee”, “discomfort” and “pain” emerged as central in participants’ evocations, highlighting both the physical symptoms and the associated emotional and social repercussions. The central core of the representations, with terms such as “urine” and “pee”, reflects the immediate and concrete perception of UI as a condition that interferes with urinary control. This connection reinforces the relevance of UI as a real and recurrent problem, as shown by recent studies^(2,17)^.

The physical impact of UI goes beyond urine loss, including complications such as urinary tract infections, burning, and discomfort, often exacerbated in cases of severe UI. Moreover, urinary urgency, reported as an experience of extreme pressure and discomfort, is often associated with the fear of leaking at inopportune times, limiting women’s routine and social activities^(18)^.

Furthermore, the data highlight the connection between “discomfort” and “pain,” two elements frequently cited in the central core. These symptoms not only affect physical health, but also exacerbate emotional distress. The discomfort caused by the sensation of continuous wetness and the need for pads were widely discussed, confirming findings that show how these characteristics aggravate the negative impact on quality of life^(4,6)^.

The results of this study revealed that “shame” is one of the central emotional pillars, connected to feelings such as “fear”, “sadness” and “embarrassment”. These findings reflect the weight of the social stigma surrounding UI, which continues to be perceived as a humiliating and embarrassing condition. Recent literature confirms that stigma associated with UI directly affects women’s self-esteem, leading to social isolation, anxiety and, in more severe cases, depression^(1,6)^.

Furthermore, shame is compounded by cultural barriers that discourage seeking care from healthcare professionals. Many women reported avoiding discussing the problem with professionals due to fear of being judged or minimized in their complaints. Previous studies have shown that perceiving UI as a “natural event” of aging or motherhood prevents the adoption of self-care behaviors and effective treatments^(19,20)^. Thus, the normalization of UI as an “inevitable” aspect of life contributes to delays in UI diagnosis and management.

Another central element of social representation of UI is the social impact that this condition has on women’s lives. Words such as “insecurity” and “embarrassment” reveal how UI restricts social interaction, leading many women to avoid public situations or social events. These restrictions reflect the constant fear of leakages or difficulties in accessing bathrooms in a timely manner^(3,12)^.

The results also indicate that feelings of shame can be aggravated by a lack of empathetic and informative support from healthcare professionals. Inadequate treatment or downplaying of symptoms can create a negative experience during medical consultations, driving women away from treatment. Careful and respectful care is crucial to combat social stigma and promote active seeking of healthcare^(13)^.

The role of nurses is essential to promote comprehensive care, addressing patients’ physical and emotional aspects. Studies indicate that the development of educational interventions and the provision of humanized support are essential to improving the quality of life of women with UI^(21)^.

Among the intermediate elements, terms such as “muscle” and “musculature” appeared prominently, highlighting the importance of strengthening the pelvic floor muscles in managing UI. Specific exercises for this region can significantly reduce UI symptoms, especially in cases of stress incontinence^(17,21)^. However, it is alarming that, despite knowledge about these exercises, the majority of study participants reported not practicing them, highlighting a gap between knowledge and practice.

Adherence to pelvic floor strengthening is recognized as a chronic challenge, influenced by factors such as lack of adequate knowledge, absence of professional monitoring and low perception of the effectiveness of treatment by women^(12,13)^. The literature indicates that structured educational strategies, combined with continuous support from healthcare professionals, are essential to increase adherence to this practice^(9)^. The inclusion of pelvic floor rehabilitation programs in the primary care services of the Brazilian Health System (In Portuguese, *Sistema Único de Saúde* SUS), combined with the training of nurses to provide detailed guidance during gynecological and prenatal consultations, could strengthen the implementation of this approach^(22)^. Furthermore, interventions that use technology, such as pelvic training applications and telemonitoring by healthcare professionals, have demonstrated a positive impact on treatment adherence in different populations^(4)^. Thus, it is reinforced that, in addition to awareness, structuring continuous and accessible support can optimize the effects of strengthening the pelvic floor and minimize the negative impacts of UI on women’s quality of life.

The findings of this study highlight the importance of developing public health strategies aimed at preventing UI, especially with regard to the inclusion of pelvic floor assessment as part of the prenatal care protocol in SUS by the Ministry of Health. Considering that UI is frequently associated with pregnancy and childbirth, implementing this assessment would allow for the early detection of risk factors and guidance on preventive measures, such as practice of exercises to strengthen pelvic floor during pregnancy and postpartum. To make this initiative viable, it is essential that healthcare professionals, especially nurses, receive specific training to perform this assessment and offer appropriate guidance. Therefore, it is recommended that academic training in nursing include this practice in the curriculum of undergraduate courses, ensuring that future generalist professionals also develop clinical skills for UI prevention and management, not only specialists. Thus, this study highlights the need to integrate the UI approach into maternal and child health policies, promoting evidence-based interventions that positively impact women’s quality of life.

In addition to prevention, it is essential that UI treatment programs be more accessible and incorporated in a structured manner into SUS care. This includes expanding the offer of conservative therapies, such as pelvic screening, as well as making devices such as pessaries available for women with prolapse, ensuring effective alternatives for managing the condition. Moreover, it is essential to increase the availability of corrective surgeries in public services, ensuring that women with more severe conditions have timely access to procedures that can restore their functionality and well-being. These measures are essential to reduce the physical, emotional and social impacts of UI, promoting a significant improvement in the quality of life of women affected by the condition.

The clinical role of nurses in managing UI should be focused on encouraging the adoption of healthy habits, such as weight control, regular physical activity and a balanced diet, factors that help reduce UI symptoms, as well as encourage adherence to behavioral therapies, such as bladder training and programmed urination techniques, to improve urinary health^(22)^.

An underrepresented but crucial aspect is the impact of UI on women’s sex lives. Recent studies show that UI can compromise physical intimacy, causing sexual dissatisfaction and even marital conflict^(4,6)^. The feeling of lack of control during sexual intercourse, combined with the fear of leakage, contributes to the loss of confidence and pleasure. Addressing these impacts in UI clinical management is essential to ensure comprehensive care.

The role of nurses in the management of female sexual dysfunctions is essential to promote comprehensive health, considering their coexistence with conditions such as UI. Nurses can provide health education, identify associated factors and guide sexual communication strategies. Emotional support during consultations reduces stigma and encourages treatment. Nursing intervention should include biopsychosocial assessment, focusing on functional rehabilitation, self-esteem and sexual well-being^(23)^. Nurses can also provide guidance on vaginal lubricants, positions that minimize pressure on the bladder, use of vaginal dilators, and referrals to specialized professionals, such as sexologists, when necessary. In this way, nurses play an essential role in promoting the sexual health of these women, contributing to improving patients’ quality of life and emotional well-being.

Although there are previous studies on UI and its impact on women’s lives, this study innovates by using SRT to deepen the understanding of how this condition is perceived in the collective female imagination, providing concrete support for enterostomal nurses’ practice. The identification of the central and peripheral elements of social representations of UI allows these professionals to structure more targeted and effective care approaches, considering not only clinical aspects, but also the emotional and social barriers that influence adherence to treatment. Moreover, the findings highlight the need to systematically incorporate pelvic floor assessment and guidance on conservative UI management strategies into nurses’ work, especially in the context of enterostomal therapy, where specialized care can prevent complications and promote a better quality of life. Thus, the study contributes evidence that reinforces the importance of nurses’ role on the front line of care for women with UI, pointing out ways to improve clinical practice and strengthen public policies focused on the topic.

The findings may also help improve nurses’ clinical practice in managing UI, especially when considering social representations that influence adherence to treatment. Understanding that feelings such as shame, discomfort, and fear are central to the perception of UI allows nurses to adopt more sensitive and humanized approaches, creating a welcoming environment that favors dialogue and adherence to therapeutic interventions. Additionally, the predominance of reports associating UI with lack of control and limitations in daily life reinforces the need for screening protocols to identify cases early and offer individualized guidance.

In the context of SUS, these data can support the inclusion of specific guidelines in nursing care, such as performing pelvic floor assessments during gynecological and prenatal consultations, implementing educational groups to provide guidance on perineal exercises and behavioral strategies, in addition to structured referrals for conservative or surgical treatments, depending on the severity of the case. Thus, the results of this study not only reinforce the relevance of UI as a public health problem, but also provide an awakening for the structuring of more effective and patient-centered care.

Despite the significant contributions of this manuscript to the understanding of social representations of UI among Brazilian women, it is essential to recognize its limitations: the use of self-administered questionnaires may introduce response bias; a cross-sectional study design captures only a specific moment in participants’ perceptions and experiences; and although the analysis tangentially addressed the impacts of UI on women’s sexual lives, this aspect was not explored in depth. The literature reveals that UI has significant implications for intimacy and sexual satisfaction, which could have been investigated in greater detail through specific instruments or questions.

Therefore, it is suggested that future research be conducted, such as longitudinal studies to investigate the evolution of social representations of UI over time. It is also recommended to incorporate the perspectives of healthcare professionals, family members and partners, in order to provide a holistic view of the impact of UI. It is also proposed to explore in more depth the implications of UI on sexual life, using both qualitative and quantitative methods.

## CONCLUSION

UI is a complex health condition that impacts several dimensions of life, imposing negative representations in both physical and emotional aspects, as evidenced by the words evoked. Symptoms go beyond physical limits, such as pain and discomfort, also affecting mental health through negative feelings, including shame, embarrassment, fear and insecurity, which, in turn, have repercussions on the self-esteem of women living with the condition.

## Data Availability

The complete data set supporting the results of this study has been made available in SciELO Data and can be accessed at DOI 10.17605/OSF.IO/79C2U.

## References

[1] Abushamma F, Mansour A, Nassar R, Badran H, Abu Alwafa R, Ktaifan M (2024). Prevalence, risk factors, and impact on quality of life due to urinary incontinence among Palestinian women: a cross-sectional study. Cureus.

[2] Souza TR, Domingos TMR, Moreira SD, Machado E, Rabelo NNF, Silva Ferreira G (2023). Incontinência urinária em mulheres: definição, etiologia e fatores de risco. Rev Iberoam Hum Cienc Educ.

[3] Ramírez JA, Tirado PO, Samur CS, Gamboa CV, Oliveres XC (2023). Health-related quality of life in women aged 20-64 years with urinary incontinence. Int Urogynecol J Pelvic Floor Dysfunct.

[4] Shabani F, Montazeri M, Alizadeh A, Bani S, Hassanpour S, Nabighadim M (2023). The relationship between urinary incontinence with sexual function and quality of life in postmenopausal women. Post Reprod Health.

[5] Oliveira LGP, Tavares ATDVB, Amorim TV, Paiva ADCPC, Salimena AMO (2020). Impact of urinary incontinence on women’s quality of life: an integrative literature review. Rev Enferm UERJ.

[6] Cox JM, Sánchez-Polán M, Mota P, Barakat R, Nagpal TS (2023). A scoping review exploring stigma associated with postpartum urinary incontinence. Int Urogynecol J Pelvic Floor Dysfunct.

[7] Burzynski B, Gibala P, Soltysiak-Gibala Z, Jurys T, Przymuszala P, Rzymski P (2022). How incontinence affects sexual activity in Polish women: results from a cross-sectional study. Int J Environ Res Public Health.

[8] O’Connell KA, Newman DK, Palmer MH (2023). When did they start? Age of onset of toileting behaviors and urinary cues as reported by older women. Womens Health Rep.

[9] Silva JG, Santos JL, Souza AI, Costa MF, Ribeiro FS, Almeida T (2021). Nursing care in the prevention and management of urinary incontinence: an integrative review. Estima: Braz J Enterostomal Ther.

[10] Moscovici S (1978). A representação social da psicanálise.

[11] Abric JC, Moreira ASP, Oliveira DC (2000). A abordagem estrutural das representações sociais.

[12] Vasconcelos CTM, Firmiano MLV, Oriá MOB, Vasconcelos No JA, Saboia DM, Bezerra LRPS (2019). Women’s knowledge, attitude, and practice related to urinary incontinence: systematic review. Int Urogynecol J Pelvic Floor Dysfunct.

[13] Roman P, Spinelli V, Gauer APM, Fiório FB, Mucke AC, Azzi VJB (2022). Prevalence and factors associated with urinary incontinence in women farmers. Fisioter Mov.

[14] Abric JC, Abric JC (2007). La recherche du noyau central et de la zone muette des représentations sociales.

[15] Abric JC (1994). Pratiques sociales et représentations.

[16] Vergès P (1992). L’évocation de l’argent: une méthode pour la définition du noyau central d’une représentation. Bull Psychol.

[17] Rodríguez-Almagro J, Hernández Martínez A, Martínez-Vázquez S, Peinado Molina RA, Bermejo-Cantarero A, Martínez-Galiano JM (2024). A qualitative exploration of the perceptions of women living with pelvic floor disorders and factors related to quality of life. J Clin Med.

[18] Soysal P, Veronese N, Ippoliti S, Pizzol D, Carrie AM, Stefanescu S (2023). The impact of urinary incontinence on multiple health outcomes: an umbrella review of meta-analyses of observational studies. Aging Clin Exp Res.

[19] Toye F, Barker KL (2020). A meta-ethnography to understand the experience of living with urinary incontinence: “Is it just part and parcel of life?. BMC Urol.

[20] Hu JC, Ding YQ, Pang HY, Yu CQ, Sun DJY, Pei P (2024). Prevalence of urinary incontinence in middle-aged and elderly adults in 10 areas in China. Chin J Epidemiol.

[21] Tavares D, Carneiro C (2023). The role of nursing in promoting comprehensive health care: a systematic review. Texto Contexto Enferm.

[22] Assis GM, Coelho MMF, Rosa TS, Oliveira FF, Silva CPC, Brito MLP (2023). Proposal for a clinical protocol for the conservative treatment of urge urinary incontinence. Estima: Braz J Enterostomal Ther.

[23] Moura LC, Andrade MF, Alves TB, Oliveira DS (2022). Nursing care and its importance in addressing female sexual dysfunction: an integrative review. Rev Bras Enferm.

