# Supplementary material for: Structural analysis of women’s social representations of urinary incontinence
Source: Rev Esc Enferm USP. 2025 Jul 7;59:e20240433. doi: 10.1590/1980-220X-REEUSP-2024-0433en (PMC12245391; doi:10.1590/1980-220X-REEUSP-2024-0433en)
Supplement: Translation

# Translation: Análise estrutural das representações sociais de mulheres sobre a incontinência urinária

**DOI:** 10.1590/1980-220X-REEUSP-2024-0433pt

**Authors:** Manuela de Mendonça Figueirêdo Coelho, Amanda Holanda de Mesquita, Beatriz Alves de Oliveira, Camila Barroso Martins, Riksberg Leite Cabral, Mônica Oliveira Batista Oriá, Mariana Cavalcante Martins, Janaína Fonseca Victor Coutinho, Fabiane do Amaral Gubert

**Affiliations:** 1 Universidade Federal do Ceará, Fortaleza, Ceará, Brasil.; 2 Prefeitura Municipal de Maracanaú, Maracanaú, Ceará, Brasil.

**Keywords:** Incontinência Urinária, Estomaterapia, Representação Social, Saúde da Mulher

## Abstract

**Objetivo:**

Avaliar a estrutura das representações sociais da incontinência urinária entre mulheres brasileiras.

**Método:**

Estudo transversal, que utilizou questionários semiestruturados aplicados *online* entre setembro de 2021 e junho de 2022. Participaram 796 mulheres brasileiras. Análises léxicas e de similaridade foram realizadas por meio dos *softwares* EVOC 2000^®^ e IRAMUTEQ^®^. Palavras evocadas foram organizadas com base em frequência e hierarquia de evocação.

**Resultados:**

As análises indicaram que as palavras “urina”, “vergonha”, “xixi”, “desconforto”, “dor”, “incômodo”, “vazamento de urina”, “infecção”, “bexiga”, “ardência”, “escapada”, “medo”, “molhado”, “problema” e “doença” estavam no núcleo central das representações sociais sobre a incontinência urinária. Esses termos foram frequentemente mencionados e apareceram prioritariamente nas respostas. Além do impacto físico, a incontinência urinária foi associada a isolamento social, ansiedade e efeitos negativos na saúde sexual.

**Conclusão:**

A incontinência urinária afeta diversos aspectos da vida das mulheres, impondo representações negativas tanto nas dimensões físicas quanto nas emocionais. Este estudo evidenciou a urgência de desestigmatizar a condição e promover abordagens de cuidado mais abrangentes que considerem as complexidades dessas representações sociais.

## INTRODUÇÃO

A incontinência urinária (IU) é uma condição de saúde pública global que afeta entre 20% e 45% das mulheres, dependendo do grupo populacional e dos critérios diagnósticos utilizados^(1)^. Caracterizada pela perda involuntária de urina, impacta múltiplas dimensões da vida feminina, abrangendo aspectos físicos, emocionais e sociais.

Pode ser classificada em três tipos principais: IU de esforço, associada a atividades que aumentam a pressão intra-abdominal; IU de urgência, vinculada à hiperatividade do músculo detrusor; e IU mista, que combina características de IU de esforço e IU de urgência, resultando em maior severidade dos sintomas. A condição é frequentemente negligenciada e estigmatizada, o que agrava os desafios para o diagnóstico precoce e o tratamento adequado^(2)^.

Os impactos da IU vão muito além das limitações físicas, como infecções urinárias, mobilidade reduzida e desconforto. Estudos mostram que a condição está associada à ansiedade, depressão e isolamento social, que afetam profundamente a qualidade de vida e a saúde mental das mulheres^(3,4,5)^. Além disso, muitas mulheres evitam discussões sobre a IU, não buscam tratamento ou minimizam sua condição devido ao estigma e às barreiras culturais, exacerbando sentimentos de vergonha e constrangimento^(6)^. Na esfera sexual, a IU pode comprometer a intimidade física, resultando em insatisfação sexual e deterioração das relações conjugais^(7,8)^.

A prática de enfermagem é essencial na prevenção e no manejo da IU em mulheres, contribuindo para a identificação precoce dos sintomas e a implementação de intervenções eficazes. Os cuidados incluem a educação sobre exercícios para o assoalho pélvico, orientação sobre hábitos miccionais e suporte emocional para mitigar os impactos psicossociais da IU, promovendo a qualidade de vida das pacientes^(9)^.

A especialidade de estomaterapia, reconhecida como uma área avançada da prática de enfermagem, é amplamente capacitada para o cuidado de condições como a IU. Os enfermeiros estomaterapeutas possuem formação especializada para abordar as demandas complexas associadas à IU, incluindo o manejo clínico, a prevenção de complicações e o suporte emocional às pacientes.

Embora a literatura médica e epidemiológica aborde a IU, a forma como as mulheres atribuem significados à IU e interpretam suas consequências ainda é incipiente, embora seja fundamental para a compreensão do comportamento diante da doença e da adesão aos tratamentos. A Teoria das Representações Sociais (TRS), proposta por Moscovici^(10)^ e ampliada por Abric^(11)^, fornece uma estrutura analítica robusta para explorar os significados atribuídos a condições de saúde, como a IU, e suas implicações nas práticas sociais.

A TRS postula que as representações sociais são formas de conhecimento construídas e compartilhadas coletivamente, resultantes de experiências cotidianas e interações sociais. Essas representações cumprem duas funções primordiais: tornar familiar o que é desconhecido e orientar comportamentos. A abordagem estrutural da teoria^(11)^ sugere que as representações sociais possuem um núcleo central, composto por elementos estáveis e amplamente compartilhados, e um sistema periférico, mais flexível e adaptável ao contexto. No caso da IU, o núcleo central das representações pode estar associado a sentimentos de vergonha e desconforto, enquanto o sistema periférico pode refletir experiências mais subjetivas, como estratégias de enfrentamento e percepções individuais.

A relevância de explorar as representações sociais da IU está em compreender como as mulheres percebem e vivenciam essa condição. As representações refletem a experiência subjetiva da IU e influenciam as práticas sociais, desde a busca por cuidados clínicos até a discussão da condição em contextos privados e públicos. Contudo, há uma lacuna na literatura atual, com poucos estudos focando na IU sob essa perspectiva, especialmente no Brasil, em que fatores culturais e sociais têm papel relevante no manejo dessa condição^(12,13)^.

O presente estudo busca preencher essa lacuna, oferecendo uma análise detalhada das representações sociais da IU entre mulheres brasileiras. A utilização da TRS permite identificar os elementos centrais e periféricos dessas representações, fornecendo subsídios para a formulação de estratégias de saúde pública que abordem as necessidades emocionais, sociais e físicas das mulheres afetadas pela IU. Além disso, os resultados deste estudo podem contribuir diretamente para a prática de enfermagem, orientando os enfermeiros no desenvolvimento de intervenções que promovam educação em saúde, prevenção de complicações e suporte emocional às mulheres. Essa perspectiva fortalece o papel do enfermeiro como agente de transformação no cuidado integral à saúde feminina. Assim, este estudo tem como objetivo avaliar a estrutura das representações sociais da IU entre mulheres brasileiras.

## MÉTODO

### Desenho do Estudo

Esta investigação adota uma abordagem qualitativa com arcabouço teórico-metodológico norteado pela TRS, com ênfase em sua vertente estrutural proposto por Moscovici^(10)^ e Abric^(11)^. No âmbito da TRS, postula-se que a expressão coletiva se estrutura em torno de um núcleo central, que encapsula a memória social, e de elementos periféricos, que refletem o contexto imediato e adaptável. Essa configuração permite uma compreensão aprofundada do objeto de estudo e das relações que os indivíduos estabelecem com a realidade percebida, bem como sua integração no universo cognitivo. Assim, a teoria busca elucidar a conexão entre sujeito e objeto, delineando representações sociais enraizadas no senso comum. A organização das informações deste manuscrito foi pautada nas recomendações do *COnsolidated criteria for REporting Qualitative research*.

A abordagem estrutural da TRS caracteriza-se por apresentar as representações sociais como um sistema dual. O núcleo central é composto por um conjunto restrito de ideias estáveis e amplamente compartilhadas pelos membros do grupo, demonstrando resistência a mudanças. Já o sistema periférico, mais flexível e sensível ao contexto imediato, deriva seu significado e função da relação com o núcleo central^(11,14)^. O sistema periférico desempenha múltiplas funções, incluindo a concretização do núcleo central por meio de posicionamentos ou condutas, a prescrição de comportamentos e a modulação personalizada. Esta última permite que uma representação se manifeste de formas aparentemente distinta, refletindo apropriações individuais ou contextos específicos, sempre em consonância com o núcleo central^(10,11)^.

### Local, População, Critérios de Seleção

Os dados foram coletados entre setembro de 2021 e junho de 2022, abrangendo um universo de mulheres brasileiras, que representam mais da metade da população nacional. A coleta ocorreu por conveniência, resultando na participação de 796 mulheres, que responderam a um questionário semiestruturado disponibilizado *online*. O convite para participar da pesquisa foi divulgado por meio de diversas plataformas de mídia social, incluindo grupos direcionados a mulheres de diferentes regiões do Brasil. Além disso, foi solicitado que as participantes compartilhassem o *link* da pesquisa com outras mulheres em suas listas de contato, ampliando o alcance da coleta.

As participantes potenciais, definidas como mulheres brasileiras maiores de 18 anos, receberam informações detalhadas sobre o estudo e que demonstaram interesse em participar da pesquisa mediante resposta à chamada veiculada em redes sociais ou *link* recebido por outros participantes. Além disso, receberam explicações sobre o objetivo da pesquisa, forma de participação. Após aceite, todas assinaram o Termo de Consentimento Livre e Esclarecido e foi *link* para acesso ao questionário.. O questionário incluía dados sociodemográficos, clínicos e obstétricos, além da questão central “Diga o que te vem imediantemaente à cabeça, imediatamente, quando você pensa em IU”, com espaços para a escrita de até três palavras ou expressões por ordem de surgimento. Foram excluídas as mulheres que não apresentaram as três evocações solicitadas sobre a IU.

### Análise de Dados

Os dados sociodemográficos, clínicos e obstétricos foram consolidados no *Excel* ^®^ e exportados para análise no *Statistical Package for the Social Sciences*, versão 23.0, sendo apresentados por meio de estatística descritiva, com frequências absolutas, relativas e desvios padrões.

Para realizar uma triangulação de dados do *corpus* textual, os dados qualitativos provenientes das evocações sobre o que as mulheres pensavam da IU foram submetidos a análises lexicais e de conteúdo. As palavras registradas foram exportadas para arquivos no formato *Word* ^®^, codificadas e padronizadas quanto às flexões de gênero e número, e, posteriormente, salvas em formato .txt, compatível com o *software Ensemble de Programmes Permettant l’Analyse des Évocations* (EVOC 2000^®^). Esse *software* é especializado na construção do Quadro de Quatro Casas, que representa os núcleos centrais e intermediários da representação.

Consideraram-se a frequência das palavras e a Ordem Média de Evocação (OME) por meio dos seguintes parâmetros: frequência mínima de 05; intermediária de 10; e Rang de 2,5^(15)^. O EVOC 2000^®^ gerou o Quadro de Quatro Casas, que organiza as evocações em quatro zonas com base na frequência e na OME:

- Núcleo central (quadrante superior esquerdo): inclui termos de alta frequência e baixa OME, representando os elementos mais estáveis e importantes para o grupo;
- Primeira periferia (quadrante superior direito): compreende elementos de alta frequência e maior OME, indicando relevância menor no imaginário imediato;
- Zona de contraste (quadrante inferior esquerdo): contém palavras de baixa frequência e evocadas rapidamente, refletindo ideias relevantes para subgrupos específicos;
- Segunda periferia (quadrante inferior direito): representa elementos de baixa frequência e alta OME, considerados menos importantes ou marginais na representação social.

Posteriormente, a Classificação Hierárquica Descendente foi realizada no *Interface de R pour les Analyses Multidimensionnelles de Textes et de Questionnaires* (IRAMUTEQ^®^), segmentando o *corpus* textual em classes hierarquicamente organizadas, destacadas no dendrograma. Cada classe é composta por palavras com alto valor de ^2^, indicando forte associação à categoria. A estrutura hierárquica permite identificar relações temáticas entre os elementos, revelando padrões emergentes no *corpus* textual.

A análise de similitude, também realizada no IRAMUTEQ^®^, identificou as relações de co-ocorrência entre os termos evocados. Os resultados foram representados graficamente por uma árvore de similitude^(16)^, em que:

- Nós principais: representam os termos mais frequentemente conectados, como “vergonha” ou “dor”;
- Conexões: a espessura das arestas indica a intensidade da associação entre os termos;
- Ramos secundários: identificam subgrupos de palavras conectadas, ajudando a interpretar nuances das representações sociais^(16)^.

### Aspectos Éticos

O estudo foi aprovado pelo Comitê de Ética em Pesquisa sob o Parecer nº 4.270.415. Todas as participantes assinaram o Termo de Consentimento Livre e Esclarecido, garantindo sigilo e voluntariedade. Os dados desta pesquisa estão disponíveis no OSF *Home* por meio do *link* https://osf.io/79c2u/.

## RESULTADOS

As mulheres que participaram do estudo tinham em média 34,6 anos (DP = 12,4), com 333 (43,3%) brancas, com companheiro (n = 482, 60,8%) e média de peso de 69,8 kg (DP = 23). Entre as participantes, 531 (66,7%) trabalhavam; 473 (59,4%) estudavam; 202 (25,4%) tinham ensino superior incompleto; 188 (23,6%) ensino superior e especialização; e 174 (21,9%) tinham apenas ensino superior.

No tocante às características clínicas e obstétricas, 371 (46,6%) estiveram grávidas, com média de 0,96 (DP = 1,2) gestações e média de 0,83 (DP = 1,1) partos normais. No momento da pesquisa, 82,8% (n = 659) referiram estar em período reprodutivo. Em relação ao conhecimento sobre a existência de exercícios para o fortalecimento do assoalho pélvico, 673 (85,5%) afirmaram conhecer a existência da prática de fortalecimento do assoalho pélvico, mas 604 (75,9%) não praticam os exercícios.

Mediante as 3.533 evocações, construiu-se o Quadro de Quatro Casas (Quadro 1).

**Quadro 1:** Estrutura da representação social de mulheres sobre incontinência urinária – Fortaleza, Ceará, Brasil, 2022.

Elementos centrais			Elementos intermediários		
Frequência ≥ 10		Rang < 2,5	Frequência ≥ 10		Rang > 2,5
Evocação	Freq*	Rang	Evocação	Freq*	Rang
Urina	148	1,574	Tratamento	19	2,737
Vergonha	128	2,148			
Xixi	128	1,414			
Desconforto	113	1,593			
Dor	103	1,650			
Incômodo	83	1,892			
Perda de urina	69	1,304			
Infecção	63	1,905			
Bexiga	37	1,946			
Ardor	32	1,969			
Escape	29	1,483			
Medo	29	2,345			
Molhada	27	2,444			
Problema	25	2,240			
Doença	23	2,348			
**Elementos intermediários**			**Elementos periféricos**		
**Frequência < 09 ≥ 05**		**Rang < 2,5**	**Frequência < 09 ≥ 05**		**Rang > 2,5**
**Evocação**	Freq*	**Rang**	**Evocação**	Freq*	**Rang**
Tosse	09	2,444	Cheiro	06	2,500
Ansiedade	08	2,375	Cura	06	2,667
Espontânea	06	2,333	Estresse	06	3,000
Frouxa	08	2,125	Musculatura	06	2,833
Incapacidade	08	2,125	Absorvente	05	2,800
Urgência	08	2,000	Cirurgia	05	2,600
Disfunção	07	2,000	Higiene	05	2,600
Pressa	05	2,000	Médico	05	2,600
Ruim	05	2,000	Remédio	05	2,800
Bexiga baixa	09	1,889			
Músculos	07	1,857			
Sofrimento	05	1,8			
Preocupação	07	1,714			
Gravidez	06	1,333			

Nota: *Frequência.

Os resultados do Quadro de Quatro Casas destacaram que o núcleo central das representações sociais da IU é composto por 15 vocábulos de alta frequência e baixa ordem OME. Entre eles, palavras como “urina” (148 vezes; OME 1,574), “vergonha” (128; OME 2,148), “xixi” (128; OME 1,414), “desconforto” (113; OME 1,593) e “dor” (103; OME 1,650) refletem tanto os sintomas físicos quanto as emoções negativas associadas à condição. Esses termos reforçam a percepção da IU como uma experiência física desconfortável e emocionalmente desgastante. A palavra “medo” (29; OME 2,345) emerge como um elemento relevante conectado ao temor de situações embaraçosas. Palavras associadas à dimensão física, como “infecção” (63; OME 1,905) e “escape” (29; OME 1,483), também aparecem com destaque, refletindo preocupações com as consequências clínicas da IU.

Na primeira periferia, identificaram-se termos relacionados a soluções potenciais, como “tratamento” (19; OME 2,737). Já na segunda periferia e na zona de contraste, palavras como “ansiedade” (8; OME 2,375), “pressa” (5; OME 2,000) e “sofrimento” (5; OME 1,800) ilustram percepções mais subjetivas e específicas de subgrupos.

Na análise da Classificação Hierárquica Descendente, o *corpus* foi constituído por 796 textos, separados em 794 segmentos de texto (STs), com aproveitamento de 691 STs (87,03%). Evidenciaram-se 3.533 ocorrências (palavras, formas ou vocábulos). O conteúdo analisado foi categorizado em três classes (Figura 1):

- Classe 1 – Problema real (40,96%): inclui palavras como “urina” (f = 107; χ^2^ = 86,43), “xixi” (f = 89; χ^2^ = 55,95) e “bexiga” (f = 47; χ^2^ = 51,31), confirmando a relevância dos sintomas centrais na vivência das participantes.
- Classe 2 – Sentimentos gerados pela perda de urina (14,76%): abrange termos como “vergonha” (f = 104; χ^2^ = 151,75), “desconforto” (f = 87; χ^2^ = 101,64) e “medo” (f = 30; χ^2^ = 42,12), destacando o impacto emocional e social da IU.
- Classe 3 – Repercussões físicas (44,28%): foca nas consequências clínicas e sintomas, como “dor” (f = 69; χ^2^ = 260,48), “infecção” (f = 31; χ^2^ = 125,44) e “ardência” (f = 22; χ^2^ = 46,52), que impactam a qualidade de vida das mulheres.

**Figura 1:** Classificação hierárquica descendente das evocações das mulheres sobre incontinência urinária – Fortaleza, Ceará, Brasil, 2022.

A interlocução das classes converge quando a classe 1 discute a incontinência como um problema real, gerador de sentimentos e repercussões físicas que, segundo a avaliada, têm conotação negativa para a vida da mulher. Houve confluência de vocábulos apresentados nas três classes e no possível núcleo central das representações sociais.

Para melhor explorar os materiais coletados, foi realizada uma análise de similitude. Por meio da análise baseada na teoria dos grafos, foi possível identificar as ocorrências textuais entre as palavras e as indicações da conexidade entre elas, auxiliando na identificação da estrutura do conteúdo de um *corpus* textual. A análise de similitude das evocações produziu a árvore máxima (Figura 2).

**Figura 2:** Análise de similitude das evocações das mulheres sobre incontinência urinária – Fortaleza, Ceará, Brasil, 2022.

A análise confirma os núcleos da representação apontadas no Quadro 1 e a interligação entre as características representativas da IU, como “xixi”, bem como os sentimentos apontados pelas mulheres, como “vergonha” e “desconforto”, além do léxico “dor”, como repercussão física.

O termo “urina” está conectado a “escape”, “perda” e “vazamento”, reforçando a associação com a perda do controle urinário e representa uma percepção imediata e concreta da IU, sendo amplamente conectado a palavras que descrevem os sintomas físicos e os desafios associados ao controle urinário. Esse nó reflete a manifestação central da condição, destacando-se pela simplicidade e universalidade no vocabulário das mulheres.

O nó “desconforto” está conectado aos vocábulos “molhado”, “insegurança”, “ardência”, “constrangimento”, “vazamento”, ansiedade”, entre outros. Ocupa lugar central na árvore de similaridade, representando tanto as dimensões físicas quanto as emocionais da experiência com a IU. Conecta-se a outros termos que refletem os impactos diretos da condição sobre o cotidiano e a qualidade de vida das mulheres.

“Vergonha” relaciona-se a sentimentos como “medo”, “tristeza” e “constrangimento”, evidenciando o impacto emocional da condição. O nó “vergonha” destaca-se como um dos pilares emocionais centrais na análise de similaridade, refletindo a carga simbólica e estigmatizante associada à IU. Esse nó apresenta conexões significativas com termos como “desconforto”, “medo”, “tristeza”, “constrangimento” e “insegurança”, que juntos desenham um quadro emocional denso e multifacetado.

O nó “dor” reflete o impacto físico da IU e está vinculado a “desconforto”, “ardência”, “infecção”, “dificuldade” e “sofrimento”. Esses termos destacam as repercussões físicas que agravam as limitações diárias e afetam a qualidade de vida das mulheres (Figura 2).

## DISCUSSÃO

Os resultados deste estudo corroboram a complexidade da IU como uma condição que impacta profundamente as dimensões físicas, emocionais e sociais da vida das mulheres. A análise estrutural das representações sociais revelou elementos centrais, intermediários e periféricos que sustentam as experiências compartilhadas sobre a IU. Palavras como “urina”, “vergonha”, “xixi”, “desconforto” e “dor” emergiram como centrais nas evocações das participantes, destacando tanto os sintomas físicos quanto as repercussões emocionais e sociais associadas. O núcleo central das representações, com termos como “urina” e “xixi”, reflete a percepção imediata e concreta da IU como uma condição que interfere no controle urinário. Essa conexão reforça a relevância da IU como um problema real e recorrente, como mostram estudos recentes^(2,17)^.

O impacto físico da IU vai além da perda de urina, incluindo complicações como infecções urinárias, ardência e desconforto, frequentemente exacerbados em casos de IU severa. Além disso, a urgência urinária, relatada como uma experiência de extrema pressão e desconforto, é frequentemente associada ao medo de vazamentos em momentos inoportunos, limitando atividades rotineiras e sociais das mulheres^(18)^.

Ademais, os dados destacam a conexão entre “desconforto” e “dor”, dois elementos frequentemente citados no núcleo central. Esses sintomas não apenas afetam a saúde física, mas também exacerbam o desgaste emocional. O desconforto causado pela sensação de umidade contínua e a necessidade de absorventes foram amplamente discutidos, confirmando achados que mostram como essas características agravam o impacto negativo sobre a qualidade de vida^(4,6)^.

Os resultados do presente estudo revelaram que a “vergonha” é um dos pilares emocionais centrais, conectada a sentimentos como “medo”, “tristeza” e “constrangimento”. Esses achados refletem o peso do estigma social em torno da IU, que continua sendo percebida como uma condição humilhante e constrangedora. A literatura recente confirma que o estigma associado à IU afeta diretamente a autoestima das mulheres, levando ao isolamento social, à ansiedade e, em casos mais graves, à depressão^(1,6)^.

Além disso, a vergonha é intensificada por barreiras culturais que desestimulam a busca por atendimento com profissionais de saúde. Muitas mulheres relataram evitar discutir o problema com profissionais devido ao medo de serem julgadas ou minimizadas em suas queixas. Estudos anteriores mostraram que a percepção da IU como um “evento natural” do envelhecimento ou da maternidade impede a adoção de comportamentos de autocuidado e tratamentos eficazes^(19,20)^. Dessa forma, a normalização da IU como um aspecto “inevitável” da vida contribui para o atraso no diagnóstico e no manejo da condição.

Outro elemento central da representação social da IU é o impacto social que essa condição exerce na vida das mulheres. Palavras como “insegurança” e “constrangimento” revelam como a IU restringe a interação social, levando muitas mulheres a evitar situações públicas ou eventos sociais. Essas restrições refletem o medo constante de vazamentos ou dificuldades em acessar banheiros em tempo hábil^(3,12)^.

Os resultados também apontam que o sentimento de vergonha pode ser agravado pela falta de suporte empático e informativo de profissionais de saúde. A abordagem inadequada ou a desvalorização dos sintomas pode criar uma experiência negativa durante as consultas médicas, afastando as mulheres do tratamento. Uma atenção cuidadosa e respeitosa é crucial para combater o estigma social e promover a busca ativa por cuidados em saúde^(13)^.

A atuação do enfermeiro é essencial para promover o cuidado integral, abordando os aspectos físicos e emocionais das pacientes. Estudos indicam que o desenvolvimento de intervenções educativas e a oferta de suporte humanizado são fundamentais para melhorar a qualidade de vida das mulheres com IU^(21)^.

Entre os elementos intermediários, termos como “músculo” e “musculatura” apareceram em destaque, ressaltando a importância do fortalecimento muscular do assoalho pélvico no manejo da IU. Exercícios específicos para essa região podem reduzir significativamente os sintomas de IU, especialmente nos casos de incontinência de esforço^(17,21)^. No entanto, é alarmante que, apesar do conhecimento sobre esses exercícios, a maioria das participantes do estudo relatou não os praticar, evidenciando uma lacuna entre conhecimento e prática.

A adesão ao fortalecimento do assoalho pélvico é reconhecida como um desafio crônico, influenciado por fatores como falta de conhecimento adequado, ausência de acompanhamento profissional e baixa percepção da eficácia do tratamento por parte das mulheres^(12,13)^. A literatura aponta que estratégias educativas estruturadas, combinadas com suporte contínuo de profissionais de saúde, são fundamentais para aumentar a adesão a essa prática^(9)^. A inclusão de programas de reabilitação do assoalho pélvico nos serviços de atenção primária do Sistema Único de Saúde (SUS), aliada à capacitação dos enfermeiros para fornecer orientações detalhadas durante consultas ginecológicas e pré-natais, poderia fortalecer a implementação dessa abordagem^(22)^. Além disso, intervenções que utilizam tecnologia, como aplicativos de treinamento pélvico e telemonitoramento por profissionais de saúde, demonstraram impacto positivo na adesão ao tratamento em diferentes populações^(4)^. Dessa forma, reforça-se que, além da conscientização, a estruturação de um suporte contínuo e acessível pode otimizar os efeitos do fortalecimento do assoalho pélvico e minimizar os impactos negativos da IU na qualidade de vida das mulheres.

Os achados deste estudo evidenciam a importância de formular estratégias de saúde pública voltadas à prevenção da IU, sobretudo no que se refere à inclusão da avaliação do assoalho pélvico como parte do protocolo de atendimento pré-natal no SUS por parte do Ministério da Saúde. Considerando que a IU está frequentemente associada à gestação e ao parto, a implementação dessa avaliação permitiria a detecção precoce de fatores de risco e a orientação sobre medidas preventivas, como a prática de exercícios para o fortalecimento do assoalho pélvico durante a gestação e pós-parto. Para viabilizar essa iniciativa, é essencial que os profissionais de saúde, especialmente os enfermeiros, recebam capacitação específica para realizar essa avaliação e oferecer orientações adequadas. Assim, recomenda-se que a formação acadêmica na área da enfermagem inclua essa prática na grade curricular dos cursos de graduação, garantindo que os futuros profissionais generalista também desenvolvam habilidades clínicas para a prevenção e manejo da IU, não apenas os especialistas. Dessa forma, este estudo instiga sobre a necessidade de integrar a abordagem da IU nas políticas de saúde materno-infantil, promovendo intervenções baseadas em evidências que impactam positivamente a qualidade de vida das mulheres.

Além da prevenção, é fundamental que programas de tratamento da IU sejam mais acessíveis e incorporados de forma estruturada ao atendimento do SUS. Isso inclui a ampliação da oferta de terapias conservadoras, como trienamento pélvico, bem como a disponibilização de dispositivos como pessários para mulheres com prolapsos, garantindo alternativas eficazes para o manejo da condição. Além disso, é essencial aumentar a disponibilidade de cirurgias corretivas nos serviços públicos, assegurando que mulheres com quadros mais graves tenham acesso oportuno a procedimentos que possam restaurar sua funcionalidade e bem-estar. Essas medidas são fundamentais para reduzir os impactos físicos, emocionais e sociais da IU, promovendo uma significativa melhora na qualidade de vida das mulheres afetadas pela condição.

A atuação clínica do enfermeiro no manejo da IU deve estar voltada para o incentivo à adoção de hábitos saudáveis, como controle de peso, prática de atividades físicas regulares e alimentação balanceada, fatores que auxiliam na redução dos sintomas da IU, bem como estimulam a adesão a terapias comportamentais, como o treinamento da bexiga e técnicas de micção programada, para melhorar a saúde urinária^(22)^.

Um aspecto sub-representado, mas crucial, é o impacto da IU na vida sexual das mulheres. Estudos recentes demonstram que a IU pode comprometer a intimidade física, causando insatisfação sexual e até mesmo conflitos conjugais^(4,6)^. A sensação de falta de controle durante a relação sexual, combinada ao medo de vazamentos, contribui para a perda de confiança e prazer. Abordar esses impactos no manejo clínico da IU é essencial para garantir o cuidado integral.

A atuação do enfermeiro no manejo das disfunções sexuais femininas é essencial para promover a saúde integral, considerando sua coexistência com condições como a IU. O enfermeiro pode oferecer educação em saúde, identificar fatores associados e orientar estratégias de comunicação sexual. O apoio emocional durante consultas reduz estigmas e encoraja o tratamento. A intervenção de enfermagem deve incluir avaliação biopsicossocial, com foco na reabilitação funcional, autoestima e bem-estar sexual^(23)^. Os enfermeiros também podem atuar na orientação sobre lubrificantes vaginais, posições que minimizem a pressão sobre a bexiga, uso de dilatadores vaginais e na indicação de encaminhamentos para profissionais especializados, como sexólogos, quando necessário. Dessa forma, o enfermeiro assume um papel essencial na promoção da saúde sexual dessas mulheres, contribuindo para a melhoria da qualidade de vida e o bem-estar emocional das pacientes.

Embora existam estudos prévios sobre a IU e seu impacto na vida das mulheres, este estudo inova ao utilizar a TRS para aprofundar a compreensão sobre como essa condição é percebida no imaginário coletivo feminino, fornecendo subsídios concretos para a prática do enfermeiro estomaterapeuta. A identificação dos elementos centrais e periféricos das representações sociais da IU permite que esses profissionais estruturem abordagens de cuidado mais direcionadas e eficazes, considerando não apenas os aspectos clínicos, mas também as barreiras emocionais e sociais que influenciam a adesão ao tratamento. Além disso, os achados ressaltam a necessidade de incorporar, de forma sistemática, a avaliação do assoalho pélvico e a orientação sobre estratégias conservadoras de manejo da IU na atuação do enfermeiro, especialmente no contexto da estomaterapia, em que o cuidado especializado pode prevenir complicações e promover maior qualidade de vida. Dessa forma, o estudo contribui com evidências que reforçam a importância da atuação do enfermeiro na linha de frente da assistência às mulheres com IU, apontando caminhos para aprimorar a prática clínica e fortalecer políticas públicas voltadas ao tema.

Os achados também podem direcionar ao aprimoramento da prática clínica dos enfermeiros no manejo da IU, especialmente ao considerar as representações sociais que influenciam a adesão ao tratamento. A compreensão de que sentimentos como vergonha, desconforto e medo são centrais na percepção da IU permite que os enfermeiros adotem abordagens mais sensíveis e humanizadas, criando um ambiente de acolhimento que favoreça o diálogo e a adesão às intervenções terapêuticas. Além disso, a predominância de relatos que associam a IU à falta de controle e limitações na vida cotidiana reforça a necessidade de protocolos de triagem para identificar precocemente os casos e oferecer orientações individualizadas.

No contexto do SUS, esses dados podem embasar a inclusão de diretrizes específicas nos atendimentos de enfermagem, como a realização de avaliações do assoalho pélvico durante consultas ginecológicas e pré-natais, a implementação de grupos educativos para orientação sobre exercícios perineais e estratégias comportamentais, além do encaminhamento estruturado para tratamentos conservadores ou cirúrgicos, conforme a gravidade do caso. Dessa forma, os resultados deste estudo não apenas reforçam a relevância da IU como um problema de saúde pública, mas também oferecem um despertar para a estruturação de uma assistência mais efetiva e centrada na paciente.

Apesar das contribuições significativas deste manuscrito para o entendimento das representações sociais da IU entre mulheres brasileiras, é fundamental reconhecer suas limitações: o uso de questionários autoaplicados pode introduzir viés de resposta; estudo com desenho transversal captura apenas um momento específico das percepções e experiências das participantes; e embora a análise tenha abordado de forma tangencial os impactos da IU na vida sexual das mulheres, esse aspecto não foi explorado em profundidade. A literatura revela que a IU tem implicações significativas na intimidade e na satisfação sexual, o que poderia ter sido investigado com maior detalhamento por meio de instrumentos ou questões específicas.

Assim, sugere-se a realização de futuras pesquisas, como estudos longitudinais para investigar a evolução das representações sociais da IU ao longo do tempo. Recomenda-se, ainda, a incorporação das perspectivas de profissionais de saúde, familiares e parceiros, a fim de proporcionar uma visão holística do impacto da IU. Também se propõe a exploração mais aprofundada das implicações da IU na vida sexual, utilizando tanto métodos qualitativos quanto quantitativos.

## CONCLUSÃO

A IU é uma condição de saúde complexa, com impactos em diversas dimensões da vida, impondo representações negativas tanto no aspecto físico quanto no emocional, como evidenciado pelos vocábulos evocados. Os sintomas ultrapassam os limites físicos, como dor e desconforto, afetando também a saúde mental por meio de sentimentos negativos, incluindo vergonha, constrangimento, medo e insegurança, o que, por sua vez, repercute na autoestima das mulheres que convivem com a condição.

## Data Availability

O conjunto completo de dados que respalda os resultados deste estudo foi disponibilizado no SciELO Data e pode ser acessado em DOI 10.17605/OSF.IO/79C2U.

# Translation: Análisis estructural de las representaciones sociales de la incontinencia urinaria en las mujeres

**DOI:** 10.1590/1980-220X-REEUSP-e20240433es

**Authors:** Manuela de Mendonça Figueirêdo Coelho, Amanda Holanda de Mesquita, Beatriz Alves de Oliveira, Camila Barroso Martins, Riksberg Leite Cabral, Mônica Oliveira Batista Oriá, Mariana Cavalcante Martins, Janaína Fonseca Victor Coutinho, Fabiane do Amaral Gubert

**Affiliations:** 1 Universidade Federal do Ceará, Fortaleza, CE, Brasil.; 2 Prefeitura Municipal de Maracanaú, Maracanaú, CE, Brasil.

**Keywords:** Incontinencia Urinaria, Estomaterapia, Representación Social, Salud de la Mujer

## Abstract

**Objetivo:**

Evaluar la estructura de las representaciones sociales de la incontinencia urinaria entre mujeres brasileñas.

**Método:**

Estudio transversal, que utilizó cuestionarios semiestructurados aplicados en línea entre septiembre de 2021 y junio de 2022. Participaron 796 mujeres brasileñas. Se realizaron análisis léxicos y de similitud utilizando el software EVOC 2000^®^ e IRAMUTEQ^®^. Las palabras evocadas se organizaron según la frecuencia y la jerarquía de evocación.

**Resultados:**

Los análisis indicaron que las palabras “orina”, “vergüenza”, “pis”, “malestar”, “dolor”, “incomodidad”, “pérdida de orina”, “infección”, “vejiga”, “ardor”, “escape”, “miedo”, “mojado”, “problema” y “enfermedad” estaban en el centro de las representaciones sociales sobre la incontinencia urinaria. Estos términos se mencionaron con frecuencia y aparecieron principalmente en las respuestas. Además del impacto físico, la incontinencia urinaria se ha asociado con el aislamiento social, la ansiedad y efectos negativos en la salud sexual.

**Conclusión:**

La incontinencia urinaria afecta varios aspectos de la vida de las mujeres, imponiendo representaciones negativas tanto en la dimensión física como emocional. Este estudio destacó la urgencia de desestigmatizar la condición y promover enfoques de atención más integrales que consideren las complejidades de estas representaciones sociales.

## INTRODUCCIÓN

La incontinencia urinaria (IU) es un problema de salud pública mundial que afecta entre el 20 % y el 45 % de las mujeres, dependiendo del grupo poblacional y de los criterios diagnósticos utilizados^(1)^. Caracterizada por la pérdida involuntaria de orina, afecta múltiples dimensiones de la vida femenina, incluyendo aspectos físicos, emocionales y sociales.

Se puede clasificar en tres tipos principales: IU de esfuerzo, asociada a actividades que aumentan la presión intraabdominal; IU de urgencia, relacionada con la hiperactividad del músculo detrusor; e IU mixta, que combina características de la IU de esfuerzo y la IU de urgencia, lo que da lugar a una mayor gravedad de los síntomas. Esta afección suele descuidarse y estigmatizarse, lo que agrava los retos para su diagnóstico precoz y su tratamiento adecuado^(2)^.

Los impactos de la IU van mucho más allá de las limitaciones físicas, como las infecciones urinarias, la movilidad reducida y la incomodidad. Los estudios muestran que la afección está asociada con la ansiedad, la depresión y el aislamiento social, que afectan profundamente la calidad de vida y la salud mental de las mujeres^(3,4,5)^. Además, muchas mujeres evitan hablar sobre la IU, no buscan tratamiento o minimizan su condición debido al estigma y las barreras culturales, lo que exacerba los sentimientos de vergüenza y bochorno^(6)^. En el ámbito sexual, la IU puede comprometer la intimidad física, lo que da lugar a insatisfacción sexual y deterioro de las relaciones conyugales^(7,8)^.

La práctica de la enfermería es esencial en la prevención y el manejo de la IU en mujeres, contribuyendo a la identificación temprana de los síntomas y a la implementación de intervenciones eficaces. Los cuidados incluyen la educación sobre ejercicios para el suelo pélvico, orientación sobre hábitos miccionales y apoyo emocional para mitigar los impactos psicosociales de la IU, promoviendo la calidad de vida de las pacientes^(9)^.

La especialidad de estomaterapia, reconocida como un área avanzada de la práctica de enfermería, está ampliamente capacitada para el cuidado de afecciones como la IU. Los enfermeros estomaterapeutas tienen formación especializada para abordar las complejas demandas asociadas a la IU, incluyendo el manejo clínico, la prevención de complicaciones y el apoyo emocional a las pacientes.

Aunque la literatura médica y epidemiológica aborda la IU, la forma en que las mujeres atribuyen significados a la IU e interpretan sus consecuencias es aún incipiente, aunque fundamental para comprender el comportamiento ante la enfermedad y la adherencia a los tratamientos. La Teoría de las Representaciones Sociales (TRS), propuesta por Moscovici^(10)^ y ampliada por Abric^(11)^, proporciona un marco analítico sólido para explorar los significados atribuidos a condiciones de salud, como la IU, y sus implicaciones en las prácticas sociales.

La TRS postula que las representaciones sociales son formas de conocimiento construidas y compartidas colectivamente, resultantes de experiencias cotidianas e interacciones sociales. Estas representaciones cumplen dos funciones primordiales: familiarizar lo desconocido y orientar los comportamientos. El enfoque estructural de la teoría^(11)^ sugiere que las representaciones sociales tienen un núcleo central, compuesto por elementos estables y ampliamente compartidos, y un sistema periférico, más flexible y adaptable al contexto. En el caso de la IU, el núcleo central de las representaciones puede estar asociado a sentimientos de vergüenza e incomodidad, mientras que el sistema periférico puede reflejar experiencias más subjetivas, como estrategias de afrontamiento y percepciones individuales.

La relevancia de explorar las representaciones sociales de la IU radica en comprender cómo las mujeres perciben y viven esta afección. Las representaciones reflejan la experiencia subjetiva de la IU e influyen en las prácticas sociales, desde la búsqueda de atención médica hasta la discusión de la afección en contextos privados y públicos. Sin embargo, existe una laguna en la literatura actual, con pocos estudios que se centren en la IU desde esta perspectiva, especialmente en Brasil, donde los factores culturales y sociales desempeñan un papel relevante en el manejo de esta afección^(12,13)^.

El presente estudio busca llenar este vacío, ofreciendo un análisis detallado de las representaciones sociales de la IU entre las mujeres brasileñas. El uso de la TRS permite identificar los elementos centrales y periféricos de estas representaciones, proporcionando insumos para la formulación de estrategias de salud pública que aborden las necesidades emocionales, sociales y físicas de las mujeres afectadas por la IU. Además, los resultados de este estudio pueden contribuir directamente a la práctica de la enfermería, orientando a los enfermeros en el desarrollo de intervenciones que promuevan la educación en salud, la prevención de complicaciones y el apoyo emocional a las mujeres. Esta perspectiva fortalece el papel del enfermero como agente de transformación en la atención integral de la salud femenina. Así, este estudio tiene como objetivo evaluar la estructura de las representaciones sociales de la IU entre las mujeres brasileñas.

## MÉTODO

### Diseño del Estudio

Esta investigación adopta un enfoque cualitativo con un marco teórico-metodológico guiado por la TRS, con énfasis en su vertiente estructural propuesta por Moscovici^(10)^ y Abric^(11)^. En el ámbito de la TRS, se postula que la expresión colectiva se estructura en torno a un núcleo central, que encapsula la memoria social, y a elementos periféricos, que reflejan el contexto inmediato y adaptable. Esta configuración permite una comprensión profunda del objeto de estudio y de las relaciones que los individuos establecen con la realidad percibida, así como su integración en el universo cognitivo. Así, la teoría busca dilucidar la conexión entre sujeto y objeto, delineando representaciones sociales arraigadas en el sentido común. La organización de la información de este manuscrito se basó en las recomendaciones de los *COnsolidated criteria for REporting Qualitative research* (*Criterios consolidados para la presentación de informes de investigación cualitativa)*.

El enfoque estructural de la TRS se caracteriza por presentar las representaciones sociales como un sistema dual. El núcleo central está compuesto por un conjunto restringido de ideas estables y ampliamente compartidas por los miembros del grupo, que muestran resistencia al cambio. Por su parte, el sistema periférico, más flexible y sensible al contexto inmediato, deriva su significado y función de la relación con el núcleo central^(11,14)^. El sistema periférico desempeña múltiples funciones, entre ellas la concretización del núcleo central a través de posicionamientos o conductas, la prescripción de comportamientos y la modulación personalizada. Esta última permite que una representación se manifieste de formas aparentemente distintas, reflejando apropiaciones individuales o contextos específicos, siempre en consonancia con el núcleo central^(10,11)^.

### Lugar, Población, Criterios de Selección

Los datos se recopilaron entre septiembre de 2021 y junio de 2022, abarcando un universo de mujeres brasileñas, que representan más de la mitad de la población nacional. La recolección se realizó por conveniencia, lo que resultó en la participación de 796 mujeres, que respondieron a un cuestionario semiestructurado disponible en línea. La invitación a participar en la investigación se difundió a través de diversas plataformas de redes sociales, incluyendo grupos dirigidos a mujeres de diferentes regiones de Brasil. Además, se pidió a las participantes que compartieran el *enlace* de la investigación con otras mujeres de sus listas de contactos, ampliando así el alcance de la recolección.

Las participantes potenciales, definidas como mujeres brasileñas mayores de 18 años, recibieron información detallada sobre el estudio y mostraron interés en participar en la investigación mediante su respuesta a la convocatoria publicada en las redes sociales o al enlace recibido por otras participantes. Además, recibieron explicaciones sobre el objetivo de la investigación y la forma de participación. Una vez aceptadas, todas firmaron el Término de Consentimiento Libre y Esclarecido y se les proporcionó un enlace para acceder al cuestionario. El cuestionario incluía datos sociodemográficos, clínicos y obstétricos, además de la pregunta central «Diga lo primero que le viene a la mente, inmediatamente, cuando piensa en IU», con espacio para escribir hasta tres palabras o expresiones por orden de aparición. Se excluyó a las mujeres que no presentaron las tres evocaciones solicitadas sobre la IU.

### Análisis de Datos

Los datos sociodemográficos, clínicos y obstétricos se consolidaron en *Excel* ^®^ y se exportaron para su análisis en el *Statistical Package for the Social Sciences*, versión 23.0, y se presentaron mediante estadísticas descriptivas, con frecuencias absolutas, relativas y desviaciones estándar.

Para realizar una triangulación de los datos del *corpus* textual, los datos cualitativos procedentes de las evocaciones sobre lo que las mujeres pensaban de la IU se sometieron a análisis léxicos y de contenido. Las palabras registradas se exportaron a archivos en formato *Word* ^®^, se codificaron y se estandarizaron en cuanto a las flexiones de género y número, y posteriormente se guardaron en formato .txt, compatible con el *software Ensemble de Programmes Permettant l’Analyse des Évocations* (EVOC 2000^®^). Este *software* está especializado en la construcción del Cuadro de Cuatro Casas, que representa los núcleos centrales e intermedios de la representación.

Se consideraron la frecuencia de las palabras y el Orden Medio de Evocación (OME) mediante los siguientes parámetros: frecuencia mínima de 05; intermedia de 10; y rango de 2,5^(15)^. El EVOC 2000^®^ generó el Cuadro de Cuatro Casas, que organiza las evocaciones en cuatro zonas en función de la frecuencia y el OME:

- Núcleo central (cuadrante superior izquierdo): incluye términos de alta frecuencia y OME baja, que representan los elementos más estables e importantes para el grupo;
- Primera periferia (cuadrante superior derecho): comprende elementos de alta frecuencia y OME mayor, lo que indica una menor relevancia en el imaginario inmediato;
- Zona de contraste (cuadrante inferior izquierdo): contiene palabras de baja frecuencia y evocadas rápidamente, que reflejan ideas relevantes para subgrupos específicos;
- Segunda periferia (cuadrante inferior derecho): representa elementos de baja frecuencia y alta OME, considerados menos importantes o marginales en la representación social.

Posteriormente, se realizó la Clasificación Jerárquica Descendente en *Interface de R pour les Analyses Multidimensionnelles de Textes et de Questionnaires* (*Interfaz de R para el Análisis Multidimensional de Textos y Cuestionarios* IRAMUTEQ^®^), segmentando el *corpus* textual en clases organizadas jerárquicamente, destacadas en el dendrograma. Cada clase está compuesta por palabras con un alto valor de c^2^, lo que indica una fuerte asociación con la categoría. La estructura jerárquica permite identificar relaciones temáticas entre los elementos, revelando patrones emergentes en el corpus textual.

El análisis de similitud, también realizado en IRAMUTEQ^®^, identificó las relaciones de coocurrencia entre los términos evocados. Los resultados se representaron gráficamente mediante un árbol de similitud^(16)^, en el que:

- Nodos principales: representan los términos más frecuentemente conectados, como «vergüenza» o «dolor»;
- Conexiones: el grosor de los bordes indica la intensidad de la asociación entre los términos;
- Ramas secundarias: identifican subgrupos de palabras conectadas, lo que ayuda a interpretar los matices de las representaciones sociales^(16)^.

### Aspectos éticos

El estudio fue aprobado por el Comité de Ética en Investigación bajo el dictamen n.º 4.270.415. Todas las participantes firmaron el Término de Consentimiento Libre y Esclarecido, garantizando la confidencialidad y la voluntariedad. Los datos de esta investigación están disponibles en OSF *Home* a través del *enlace* https://osf.io/79c2u/.

## RESULTADOS

Las mujeres que participaron en el estudio tenían una media de 34,6 años (DE = 12,4), 333(43,3%) eran blancas, tenían pareja (n = 482, 60,8%) y un peso medio de 69,8 kg (DE = 23). Entre las participantes, 531(66,7%) trabajaban; 473(59,4%) estudiaban; 202(25,4%) tenían estudios superiores incompletos; 188(23,6%) tenían estudios superiores y especialización; y 174(21,9%) solo tenían estudios superiores.

En cuanto a las características clínicas y obstétricas, 371(46,6%) habían estado embarazadas, con una media de 0,96 (DE = 1,2) embarazos y una media de 0,83 (DE = 1,1) partos normales. En el momento de la investigación, el 82,8 % (n = 659) afirmaron estar en edad reproductiva. En cuanto al conocimiento sobre la existencia de ejercicios para fortalecer el suelo pélvico, 673 (85,5%) afirmaron conocer la práctica de fortalecimiento del suelo pélvico, pero 604(75,9%) no realizaban los ejercicios.

A partir de las 3533 evocaciones, se construyó el Cuadro de las Cuatro Casas (Cuadro 1).

**Cuadro 1:** Estructura de la representación social de las mujeres sobre la incontinencia urinaria – Fortaleza, Ceará, Brasil, 2022.

Elementos centrales			Elementos intermedios		
Frecuencia ≥ 10		Rang < 2,5	Frecuencia ≥ 10		Rang > 2,5
Evocación	Freq*	Rang	Evocación	Freq*	Rang
Orina	148	1,574	Tratamiento	19	2,737
Verguenza	128	2,148			
Pis	128	1,414			
Molestia	113	1,593			
Dolor	103	1,650			
Incomodidad	83	1,892			
Pérdida de orina	69	1,304			
Infección	63	1,905			
Vejiga	37	1,946			
Ardor	32	1,969			
Escape	29	1,483			
Miedo	29	2,345			
Mojada	27	2,444			
Problema	25	2,240			
Enfermedad	23	2,348			
**Elementos intermedios**			**Elementos periféricos**		
**Frecuencia < 09 ≥ 05**		**Rang < 2,5**	**Frecuencia <09 ≥ 05**		**Rang > 2,5**
**Evocación**	Freq*	**Rang**	**Evocación**	Freq*	**Rang**
Tos	09	2,444	Olor	06	2,500
Ansiedade	08	2,375	Cura	06	2,667
Espontánea	06	2,333	Estrés	06	3,000
Floja	08	2,125	Musculatura	06	2,833
Incapacidad	08	2,125	Absorbente	05	2,800
Urgencia	08	2,000	Cirugía	05	2,600
Disfunción	07	2,000	Higiene	05	2,600
Prisa	05	2,000	Médico	05	2,600
Malo	05	2,000	Remedio	05	2,800
Vejiga baja	09	1,889			
Músculos	07	1,857			
Sufrimiento	05	1,8			
Preocupación	07	1,714			
Embarazo	06	1,333			

Nota: *Freq = Frecuencia.

Los resultados del Cuadro de Cuatro Casas destacaron que el núcleo central de las representaciones sociales de la IU está compuesto por 15 palabras de alta frecuencia y bajo orden OME. Entre ellas, palabras como «orina» (148 veces; OME 1,574), «vergüenza» (128; OME 2,148), «pis» (128; OME 1,414), «malestar» (113; OME 1,593) y «dolor» (103; OME 1,650) reflejan tanto los síntomas físicos como las emociones negativas asociadas a la afección. Estos términos refuerzan la percepción de la IU como una experiencia física incómoda y emocionalmente agotadora. La palabra «miedo» (29; OME 2,345) surge como un elemento relevante relacionado con el temor a situaciones embarazosas. Las palabras asociadas a la dimensión física, como «infección» (63; OME 1,905) y «escape» (29; OME 1,483), también aparecen destacadas, lo que refleja la preocupación por las consecuencias clínicas de la IU.

En la primera periferia, se identificaron términos relacionados con posibles soluciones, como «tratamiento» (19; OME 2,737). Ya en la segunda periferia y en la zona de contraste, palabras como «ansiedad» (8; OME 2,375), «prisa» (5; OME 2,000) y «sufrimiento» (5; OME 1,800) ilustran percepciones más subjetivas y específicas de subgrupos.

En el análisis de la Clasificación Jerárquica Descendente, el *corpus* estuvo constituido por 796 textos, separados en 794 segmentos de texto (ST), con un aprovechamiento de 691 ST (87,03 %). Se evidenciaron 3533 ocurrencias (palabras, formas o vocabulario). El contenido analizado se clasificó en tres clases (Figura 1):

- Clase 1 – Problema real (40,96 %): incluye palabras como «orina» (f = 107; χ^2^ = 86,43), «pis» (f = 89; χ^2^ = 55,95) y «vejiga» (f = 47; χ^2^ = 51,31), lo que confirma la relevancia de los síntomas centrales en la experiencia de las participantes.
- Clase 2 – Sentimientos generados por la pérdida de orina (14,76%): abarca términos como «vergüenza» (f = 104; χ^2^ = 151,75), «incomodidad» (f = 87; χ^2^ = 101,64) y «miedo» (f = 30; χ^2^ = 42,12), destacando el impacto emocional y social de la IU.
- Clase 3 – Repercusiones físicas (44,28%): se centra en las consecuencias clínicas y los síntomas, como «dolor» (f = 69; χ^2^ = 260,48), «infección» (f = 31; χ^2^ = 125,44) y «ardor» (f = 22; χ^2^ = 46,52), que afectan a la calidad de vida de las mujeres.

**Figura 1:** Clasificación jerárquica descendente de las evocaciones de las mujeres sobre la incontinencia urinaria – Fortaleza, Ceará, Brasil, 2022.

El diálogo entre las clases converge cuando la clase 1 aborda la incontinencia como un problema real, generador de sentimientos y repercusiones físicas que, según la evaluada, tienen una connotación negativa para la vida de la mujer. Hubo una confluencia de términos presentados en las tres clases y en el posible núcleo central de las representaciones sociales.

Para explorar mejor los materiales recolectados, se realizó un análisis de similitud. Mediante el análisis basado en la teoría de grafos, fue posible identificar las ocurrencias textuales entre las palabras y las indicaciones de conexión entre ellas, lo que ayudó a identificar la estructura del contenido de un *corpus textual*.

El análisis de similitud de las evocaciones produjo el árbol máximo (Figura 2).

**Figura 2:** Análisis de similitud de las evocaciones de las mujeres sobre la incontinencia urinaria – Fortaleza, Ceará, Brasil, 2022.

El análisis confirma los núcleos de representación señalados en lo Cuadro 1 y la interconexión entre las características representativas de la IU, como «pis», así como los sentimientos señalados por las mujeres, como «vergüenza» e «incómodo», además del léxico «dolor», como repercusión física.

El término «orina» está conectado con «escape», «pérdida» y «goteo», lo que refuerza la asociación con la pérdida del control urinario y representa una percepción inmediata y concreta de la IU, estando ampliamente conectado con palabras que describen los síntomas físicos y los retos asociados al control urinario. Este nodo refleja la manifestación central de la afección, destacando por su simplicidad y universalidad en el vocabulario de las mujeres.

El nodo «malestar» está relacionado con los términos «mojado», «inseguridad», «ardor», «vergüenza», «goteo», «ansiedad», entre otros. Ocupa un lugar central en el árbol de similitudes, representando tanto las dimensiones físicas como emocionales de la experiencia con la IU. Se conecta con otros términos que reflejan los impactos directos de la afección en la vida cotidiana y la calidad de vida de las mujeres.

«Vergüenza» se relaciona con sentimientos como «miedo», «tristeza» y «vergüenza», lo que pone de manifiesto el impacto emocional de la afección. El nodo «vergüenza» destaca como uno de los pilares emocionales centrales en el análisis de similitud, reflejando la carga simbólica y estigmatizante asociada a la IU. Este nodo presenta conexiones significativas con términos como «malestar», «miedo», «tristeza», «vergüenza» e «inseguridad», que juntos dibujan un cuadro emocional denso y multifacético.

El nodo «dolor» refleja el impacto físico de la IU y está vinculado a «malestar», «ardor», «infección», «dificultad» y «sufrimiento». Estos términos destacan las repercusiones físicas que agravan las limitaciones diarias y afectan a la calidad de vida de las mujeres (Figura 2).

## DISCUSIÓN

Los resultados de este estudio corroboran la complejidad de la IU como una afección que afecta profundamente las dimensiones físicas, emocionales y sociales de la vida de las mujeres. El análisis estructural de las representaciones sociales reveló elementos centrales, intermedios y periféricos que sustentan las experiencias compartidas sobre la IU. Palabras como «orina», «vergüenza», «pis», «malestar» y «dolor» surgieron como centrales en las evocaciones de las participantes, destacando tanto los síntomas físicos como las repercusiones emocionales y sociales asociadas. El núcleo central de las representaciones, con términos como «orina» y «pis», refleja la percepción inmediata y concreta de la IU como una condición que interfiere en el control urinario. Esta conexión refuerza la relevancia de la IU como un problema real y recurrente, tal y como muestran estudios recientes^(2,17)^.

El impacto físico de la IU va más allá de la pérdida de orina, incluyendo complicaciones como infecciones urinarias, ardor y malestar, a menudo exacerbados en casos de IU grave. Además, la urgencia urinaria, descrita como una experiencia de presión y malestar extremos, se asocia a menudo con el miedo a las pérdidas en momentos inoportunos, lo que limita las actividades cotidianas y sociales de las mujeres^(18)^.

Además, los datos destacan la conexión entre «malestar» y «dolor», dos elementos que se mencionan con frecuencia en el núcleo central. Estos síntomas no solo afectan a la salud física, sino que también exacerban el desgaste emocional. La incomodidad causada por la sensación de humedad continua y la necesidad de usar absorbentes se han discutido ampliamente, lo que confirma los hallazgos que muestran cómo estas características agravan el impacto negativo sobre la calidad de vida^(4,6)^.

Los resultados del presente estudio revelaron que la «vergüenza» es uno de los pilares emocionales centrales, conectado a sentimientos como «miedo», «tristeza» y «bochorno». Estos hallazgos reflejan el peso del estigma social que rodea a la IU, que sigue siendo percibida como una afección humillante y vergonzosa. La literatura reciente confirma que el estigma asociado a la IU afecta directamente a la autoestima de las mujeres, lo que conduce al aislamiento social, la ansiedad y, en casos más graves, a la depresión^(1,6)^.

Además, la vergüenza se ve intensificada por barreras culturales que desalientan la búsqueda de atención con profesionales de la salud. Muchas mujeres informaron que evitan discutir el problema con profesionales por temor a ser juzgadas o minimizadas en sus quejas. Estudios anteriores han demostrado que la percepción de la IU como un «evento natural» del envejecimiento o de la maternidad impide la adopción de comportamientos de autocuidado y tratamientos eficaces^(19,20)^. De este modo, la normalización de la IU como un aspecto «inevitable» de la vida contribuye al retraso en el diagnóstico y el tratamiento de la afección.

Otro elemento central de la representación social de la IU es el impacto social que esta afección tiene en la vida de las mujeres. Palabras como «inseguridad» y «vergüenza» revelan cómo la IU restringe la interacción social, lo que lleva a muchas mujeres a evitar situaciones públicas o eventos sociales. Estas restricciones reflejan el miedo constante a las pérdidas o a las dificultades para acceder a los baños a tiempo^(3,12)^.

Los resultados también indican que el sentimiento de vergüenza puede verse agravado por la falta de apoyo empático e informativo por parte de los profesionales de la salud. El enfoque inadecuado o la desvalorización de los síntomas pueden crear una experiencia negativa durante las consultas médicas, alejando a las mujeres del tratamiento. Una atención cuidadosa y respetuosa es crucial para combatir el estigma social y promover la búsqueda activa de atención en salud^(13)^.

La actuación del enfermero es esencial para promover la atención integral, abordando los aspectos físicos y emocionales de las pacientes. Estudios indican que el desarrollo de intervenciones educativas y la oferta de apoyo humanizado son fundamentales para mejorar la calidad de vida de las mujeres con IU^(21)^.

Entre los elementos intermedios, términos como «músculo» y «musculatura» aparecieron destacados, subrayando la importancia del fortalecimiento muscular del suelo pélvico en el manejo de la IU. Los ejercicios específicos para esta región pueden reducir significativamente los síntomas de la IU, especialmente en los casos de incontinencia de esfuerzo^(17,21)^. Sin embargo, es alarmante que, a pesar del conocimiento sobre estos ejercicios, la mayoría de las participantes en el estudio informaron que no los practicaban, lo que pone de manifiesto una brecha entre el conocimiento y la práctica.

La adherencia al fortalecimiento del suelo pélvico se reconoce como un reto crónico, influido por factores como la falta de conocimientos adecuados, la ausencia de seguimiento profesional y la baja percepción de la eficacia del tratamiento por parte de las mujeres^(12,13)^. La literatura señala que las estrategias educativas estructuradas, combinadas con el apoyo continuo de profesionales de la salud, son fundamentales para aumentar la adherencia a esta práctica^(9)^. La inclusión de programas de rehabilitación del suelo pélvico en los servicios de atención primaria del Sistema Único de Salud (SUS), junto con la capacitación de los enfermeros para proporcionar orientación detallada durante las consultas ginecológicas y prenatales, podría fortalecer la implementación de este enfoque^(22)^. Además, las intervenciones que utilizan tecnología, como aplicaciones de entrenamiento pélvico y telemonitorización por parte de profesionales de la salud, han demostrado un impacto positivo en la adherencia al tratamiento en diferentes poblaciones^(4)^. De este modo, se refuerza que, además de la concienciación, la estructuración de un apoyo continuo y accesible puede optimizar los efectos del fortalecimiento del suelo pélvico y minimizar los impactos negativos de la IU en la calidad de vida de las mujeres.

Los hallazgos de este estudio evidencian la importancia de formular estrategias de salud pública orientadas a la prevención de la IU, sobre todo en lo que se refiere a la inclusión de la evaluación del suelo pélvico como parte del protocolo de atención prenatal en el SUS por parte del Ministerio de Salud. Teniendo en cuenta que la IU se asocia frecuentemente con el embarazo y el parto, la implementación de esta evaluación permitiría la detección precoz de factores de riesgo y la orientación sobre medidas preventivas, como la práctica de ejercicios para fortalecer el suelo pélvico durante el embarazo y el posparto. Para que esta iniciativa sea viable, es esencial que los profesionales de la salud, especialmente los enfermeros, reciban formación específica para realizar esta evaluación y ofrecer orientaciones adecuadas. Por lo tanto, se recomienda que la formación académica en el área de enfermería incluya esta práctica en el plan de estudios de los cursos de grado, garantizando que los futuros profesionales generalistas también desarrollen habilidades clínicas para la prevención y el manejo de la IU, y no solo los especialistas. De este modo, este estudio insta a la necesidad de integrar el enfoque de la IU en las políticas de salud maternoinfantil, promoviendo intervenciones basadas en la evidencia que tengan un impacto positivo en la calidad de vida de las mujeres.

Además de la prevención, es fundamental que los programas de tratamiento de la IU sean más accesibles y se incorporen de manera estructurada a la atención del SUS. Esto incluye la ampliación de la oferta de terapias conservadoras, como el entrenamiento pélvico, así como la disponibilidad de dispositivos como pesarios para mujeres con prolapsos, garantizando alternativas eficaces para el manejo de la afección. Además, es esencial aumentar la disponibilidad de cirugías correctivas en los servicios públicos, garantizando que las mujeres con cuadros más graves tengan acceso oportuno a procedimientos que puedan restaurar su funcionalidad y bienestar. Estas medidas son fundamentales para reducir los impactos físicos, emocionales y sociales de la IU, promoviendo una mejora significativa en la calidad de vida de las mujeres afectadas por la afección.

La actuación clínica del enfermero en el manejo de la IU debe estar orientada a fomentar la adopción de hábitos saludables, como el control del peso, la práctica de actividades físicas regulares y una alimentación equilibrada, factores que ayudan a reducir los síntomas de la IU, así como estimular la adhesión a terapias conductuales, como el entrenamiento de la vejiga y las técnicas de micción programada, para mejorar la salud urinaria^(22)^.

Un aspecto poco representado, pero crucial, es el impacto de la IU en la vida sexual de las mujeres. Estudios recientes demuestran que la IU puede comprometer la intimidad física, causando insatisfacción sexual e incluso conflictos conyugales^(4,6)^. La sensación de falta de control durante las relaciones sexuales, combinada con el miedo a las pérdidas, contribuye a la pérdida de confianza y placer. Abordar estos impactos en el manejo clínico de la IU es esencial para garantizar una atención integral.

La actuación del enfermero en el manejo de las disfunciones sexuales femeninas es esencial para promover la salud integral, teniendo en cuenta su coexistencia con afecciones como la IU. El enfermero puede ofrecer educación en salud, identificar factores asociados y orientar estrategias de comunicación sexual. El apoyo emocional durante las consultas reduce el estigma y fomenta el tratamiento. La intervención de enfermería debe incluir una evaluación biopsicosocial, centrada en la rehabilitación funcional, la autoestima y el bienestar sexual^(23)^. Los enfermeros también pueden orientar sobre lubricantes vaginales, posiciones que minimicen la presión sobre la vejiga, el uso de dilatadores vaginales y derivar a profesionales especializados, como sexólogos, cuando sea necesario. De esta manera, el enfermero asume un papel esencial en la promoción de la salud sexual de estas mujeres, contribuyendo a mejorar la calidad de vida y el bienestar emocional de las pacientes.

Aunque existen estudios previos sobre la IU y su impacto en la vida de las mujeres, este estudio innova al utilizar la TRS para profundizar en la comprensión de cómo se percibe esta afección en el imaginario colectivo femenino, proporcionando aportes concretos para la práctica del enfermero estomaterapeuta. La identificación de los elementos centrales y periféricos de las representaciones sociales de la IU permite a estos profesionales estructurar enfoques de atención más específicos y eficaces, teniendo en cuenta no solo los aspectos clínicos, sino también las barreras emocionales y sociales que influyen en la adherencia al tratamiento. Además, los hallazgos resaltan la necesidad de incorporar, de manera sistemática, la evaluación del suelo pélvico y la orientación sobre estrategias conservadoras de manejo de la IU en la actuación del enfermero, especialmente en el contexto de la estomaterapia, en el que el cuidado especializado puede prevenir complicaciones y promover una mayor calidad de vida. De este modo, el estudio contribuye con evidencias que refuerzan la importancia de la actuación de los enfermeros en la primera línea de la asistencia a las mujeres con IU, señalando caminos para mejorar la práctica clínica y fortalecer las políticas públicas orientadas al tema.

Los hallazgos también pueden orientar la mejora de la práctica clínica de los enfermeros en el manejo de la IU, especialmente al considerar las representaciones sociales que influyen en la adherencia al tratamiento. La comprensión de que sentimientos como la vergüenza, la incomodidad y el miedo son fundamentales en la percepción de la IU permite a los enfermeros adoptar enfoques más sensibles y humanizados, creando un ambiente acogedor que favorece el diálogo y la adherencia a las intervenciones terapéuticas. Además, la predominancia de informes que asocian la IU con la falta de control y las limitaciones en la vida cotidiana refuerza la necesidad de protocolos de detección para identificar precozmente los casos y ofrecer orientaciones individualizadas.

En el contexto del SUS, estos datos pueden servir de base para la inclusión de directrices específicas en la atención de enfermería, como la realización de evaluaciones del suelo pélvico durante las consultas ginecológicas y prenatales, la implementación de grupos educativos para orientar sobre ejercicios perineales y estrategias conductuales, además de la derivación estructurada a tratamientos conservadores o quirúrgicos, según la gravedad del caso. De este modo, los resultados de este estudio no solo refuerzan la relevancia de la IU como problema de salud pública, sino que también despiertan la necesidad de estructurar una asistencia más eficaz y centrada en la paciente.

A pesar de las importantes contribuciones de este manuscrito a la comprensión de las representaciones sociales de la IU entre las mujeres brasileñas, es fundamental reconocer sus limitaciones: el uso de cuestionarios autoaplicados puede introducir sesgos en las respuestas; el estudio con diseño transversal solo captura un momento específico de las percepciones y experiencias de las participantes; y aunque el análisis abordó de manera tangencial los impactos de la IU en la vida sexual de las mujeres, este aspecto no se exploró en profundidad. La literatura revela que la IU tiene implicaciones significativas en la intimidad y la satisfacción sexual, lo que podría haberse investigado con mayor detalle mediante instrumentos o preguntas específicas.

Por lo tanto, se sugiere realizar investigaciones futuras, como estudios longitudinales para investigar la evolución de las representaciones sociales de la IU a lo largo del tiempo. Se recomienda, además, incorporar las perspectivas de los profesionales de la salud, los familiares y las parejas, con el fin de proporcionar una visión holística del impacto de la IU. También se propone una exploración más profunda de las implicaciones de la IU en la vida sexual, utilizando tanto métodos cualitativos como cuantitativos.

## CONCLUSIÓN

La IU es una condición de salud compleja, con impactos en diversas dimensiones de la vida, que impone representaciones negativas tanto en el aspecto físico como en el emocional, como lo evidencian los términos evocados. Los síntomas trascienden los límites físicos, como el dolor y la incomodidad, y también afectan a la salud mental a través de sentimientos negativos, como la vergüenza, la incomodidad, el miedo y la inseguridad, lo que, a su vez, repercute en la autoestima de las mujeres que conviven con esta afección.

## Data Availability

El conjunto completo de datos que respalda los resultados de este estudio se puso a disposición en SciELO Data y se puede acceder a él em DOI 10.17605/OSF.IO/79C2U.
